# Point-Of-Care p24 Infant Testing for HIV May Increase Patient Identification despite Low Sensitivity

**DOI:** 10.1371/journal.pone.0169497

**Published:** 2017-01-06

**Authors:** Bindiya Meggi, Timothy Bollinger, Nédio Mabunda, Adolfo Vubil, Ocean Tobaiwa, Jorge I. Quevedo, Osvaldo Loquiha, Lara Vojnov, Trevor F. Peter, Ilesh V. Jani

**Affiliations:** 1 Instituto Nacional de Saúde, Maputo, Mozambique; 2 Clinton Health Access Initiative, Maputo, Mozambique; 3 Department of Mathematics and Informatics, Universidade Eduardo Mondlane, Maputo, Mozambique; Waseda University, JAPAN

## Abstract

The long delay in returning test results during early infant diagnosis of HIV (EID) often causes loss-to-follow-up prior to antiretroviral treatment (ART) initiation in resource-limited settings. A point-of-care (POC) test may help overcome these challenges. We evaluated the performance of the LYNX p24 Antigen POC test in Mozambique. 879 HIV-exposed infants under 18 months of age were enrolled consecutively at three primary healthcare clinics (PHC). Lancet heel-drawn blood was tested on-site by nurses using a prototype POC test for HIV Gag p24 antigen detection. Results of POC testing were compared to laboratory-based nucleic acid testing on dried blood spots. A comparison of the effect of sensitivity and timely test results return on successful diagnosis by POC and laboratory-based platforms was also calculated. The sensitivity and specificity of the LYNX p24 Ag test were 71.9%; (95% confidence interval [CI]: 58.5–83.0%) and 99.6% (95% CI: 98.9–99.9%), respectively. The predictive value of positive and negative tests were 93.2% (95% CI: 81.3–98.6%) and 97.9% (95% CI: 96.8–98.8%), respectively. Overall agreement was high (Cohen Kappa = 0.80; 95% CI: 0.71–0.89). Despite its lower sensitivity, the POC test had the potential to provide test results to up to 81% more patients compared to the laboratory-based test. This prototype POC p24 assay was feasible for use in PHCs but demonstrated low sensitivity for HIV detection. POC EID technologies that perform below standard recommendations may still be valuable diagnostic tools in settings with inefficient EID networks.

## Introduction

Children living with HIV continue to experience persistent treatment gaps with a coverage that is two-third of that of pregnant women (51% versus 80%) in 2015.[1] Inadequate access to early infant diagnosis (EID) is a major reason for the treatment gap in many settings [1]. The World Health Organization (WHO) recommends testing of exposed infants by six weeks of life, or at the earliest opportunity thereafter, and immediate ART initiation for all HIV-infected children under five years of age [2,3]. However, in 21 priority African countries, only 51% of HIV-exposed infants received HIV virological testing within the first two months of life in 2015 [1].

The current gold standard for EID testing, polymerase chain reaction (PCR), is a technologically complex nucleic acid test, that requires considerable infrastructure and training available at central reference laboratories and specimen transport networks to enable test access to health facilities [4]. Although several operational innovations have been made to improve the efficiency of this conventional system, the time required to transport samples, conduct the test and communicate results back to health facilities and patients remain long in many settings, resulting in high loss-to-follow-up and low ART initiation rates [5]. In Mozambique, an estimated 62% of HIV-exposed infants received EID results more than 1 month after sample collection in 2014, and this likely contributes to increased loss to follow-up, morbidity and mortality.

Point-of-care (POC) diagnostics do not require sophisticated laboratory capacity and can be operated by health care workers with limited laboratory training [3]. POC EID testing could facilitate same day testing and referral of HIV-infected infants to treatment [6,7]. Several devices for POC EID are in development, including assays that detect HIV nucleic acid or HIV Gag p24 antigen. Various studies have highlighted the utility of laboratory-based HIV Gag p24 antigen detection for adult and pediatric HIV screening [8–11]. These studies have been carried out with ultrasensitive p24 assays, which are similar in complexity to laboratory-based nucleic acid techniques in that they are time and labor intensive, and require expensive laboratory equipment, and skilled operators.

This study evaluated the diagnostic accuracy of a novel POC HIV Gag p24 Antigen Test for detection of HIV infection among HIV-exposed infants at primary health care clinics in Mozambique and estimated its impact on timely patient diagnosis.

## Materials and Methods

### Study setting and participants

Participants in this blinded cross-sectional study were tested using both POC and laboratory-based nucleic acid platforms for EID. Patient enrollment and POC testing were conducted at three public primary health care clinics located in peri-urban areas of Maputo city: 1° de Junho, 1° de Maio and Polana Caniço. These health care facilities provide routine access to EID with laboratory-based nucleic acid testing. Nurses working at these sites had been previously trained to collect dried blood spot (DBS) specimens that are routinely sent to the Instituto Nacional de Saúde (INS) reference laboratory in Maputo for testing using laboratory-based nucleic acid platforms. These health care facilities were selected based on their antenatal HIV prevalence and proximity to the INS laboratory. HIV-exposed infants presenting for routine DBS collection in existing PMTCT program, older than 28 days and younger than 18 months of age were eligible for inclusion in the study. Mothers or guardians were invited and consented to participate in the study by a study counsellor.

### Sample size

The sample size was determined assuming that the POC p24 antigen test will not be more than 95% sensitive and 99% specific for detecting HIV infection among HIV-exposed infants, with 80% power and 95% confidence intervals. Using an estimated HIV prevalence of 11% and an eligibility loss of 25%, the study would require a sample size of 885 HIV-exposed infants being enrolled to estimate the POC p24 antigen's accuracy parameters.

### Data collection

Demographic and clinical data were collected prospectively using standardized forms. Technicians conducting the laboratory-based assay were blinded to the POC test result. POC EID test results were not shared with study participants. Only laboratory-based test results were provided to study participants per national testing guidelines. Appropriate coding of samples and forms was performed to ensure confidentiality of the data. Double data entry of demographic data and POC test results was performed.

### Point of Care (POC) testing

Nurses trained to use the LYNX p24 antigen test (Northwestern University, USA) conducted onsite POC testing at the study clinics. The LYNX HIV p24 antigen test is an immunochromatographic assay that uses a pair of monoclonal antibodies to detect HIV Gag p24 antigen in heat-treated plasma. POC testing was done according to manufacturer instructions. Briefly, 80μl of fresh blood from a heel/finger stick was drawn into a blood collection tube and added immediately to the LYNX plasma separator. After 10 minutes, the plasma collection pad was plunged into the reaction tube. The reaction tube was then separated from the plasma separator and gently tapped in order to drop the pad to the bottom of the reaction tube. A buffer solution was added to the tube and then the tube was inserted in the processor device. After 11 minutes, the test strip was inserted into the reaction chamber. After a further 30 minutes, the test strip was removed from the tube and test results are interpreted immediately by the presence or absence of visually detectable gray-to-black lines. Six nurses were trained by the manufacturer or by trainers who were certified by the manufacturer prior to the start of the study. One full day training was provided to each nurse with one week of supervised testing after study initiation. A study coordinator periodically reviewed the performance of testing at the clinics to ensure adherence to test standard operating procedures and quality management.

### Standard of Care (SOC) laboratory based testing

At the same time as POC testing, nurses collected an additional 4–5 drops of whole blood onto filter paper cards (Whatman 903, GE Healthcare Biosciences, Pittsburgh, PA, USA) to create DBS specimens. Specimens were dried at room temperature before being packaged for transport to the reference laboratory. Samples were tested using the gold standard Roche COBAS AmpliPrep/COBAS TaqMan (CAP/CTM 96) HIV-1 Qualitative Test (Roche Molecular Diagnostics, Branchburg NJ, USA) or Roche Amplicor HIV-1 DNA test, version 1.5 (Roche Molecular Diagnostics, Branchburg, NJ) according to the manufacturer’s instructions. These tests detect total HIV-1 total nucleic acid (DNA and RNA) when used on whole blood samples extracted from DBS cards [11–13]. Laboratory testing was conducted by three technicians who were trained and certified to run the assays. The reference laboratory had routinely participated in and passed an EID external quality assessment program (provided by the Centers for Disease Control and Prevention, Atlanta, USA) prior to and during the study.

### Statistical methods

The sensitivity and specificity of the LYNX p24 Ag test were estimated using the laboratory-based nucleic acid test as reference. McNemar’s test with Yate’s correction was used to test the hypothesis of equality of test results between the POC and laboratory assays, and Pearson’s chi-square test to evaluate whether the sensitivity of POC EID was associated with patients characteristics such as age, mother and infant prophylaxis. Agreement between POC and SOC assays was assessed with the conditional probability of positive and negative agreement [14]. Inference was based on 95% confidence intervals for estimates of accuracy and agreement measures, and hypothesis testing with 5% significance level.

### Diagnostic yield

The performance of the LYNX p24 Ag test observed in this study was used to model its potential impact on completing the diagnosis of HIV-positive infants. The proportion of infants successfully diagnosed by the POC and laboratory tests was estimated using sensitivity and timely result return rates (timely defined as within 28 days, as per Mozambique national EID targets)[3][3]. Starting with a theoretical pool of 1,000 HIV-infected infants, the number of patients successfully receiving a timely result was estimated by multiplying sensitivity by result return rates that were varied at 5% intervals. This model provides a simplified means to compare assays with varying technical performance and result delivery efficiency that may affect successful diagnosis and subsequent linkage to care. Data on standard of care laboratory-based testing in Mozambique and the POC EID assay results from this study were used to develop the model results. Additional scenarios were added as theoretical comparisons, to show how a technically better performing POC EID assay (higher sensitivity) and an operationally better performing SOC system (more results returned) would affect potential yield.

### Ethical considerations

This study was approved by Mozambique’s National Health Bioethics Committee. Parents or legal guardians of the HIV-exposed infants were invited to participate in the study and were provided with a detailed study information sheet. A signed informed consent form was obtained for each parent or guardian that agreed to participate in the study. Patients identifying information was coded in order to maintain confidentiality.

## Results

### Patient population

**Fig 1:**
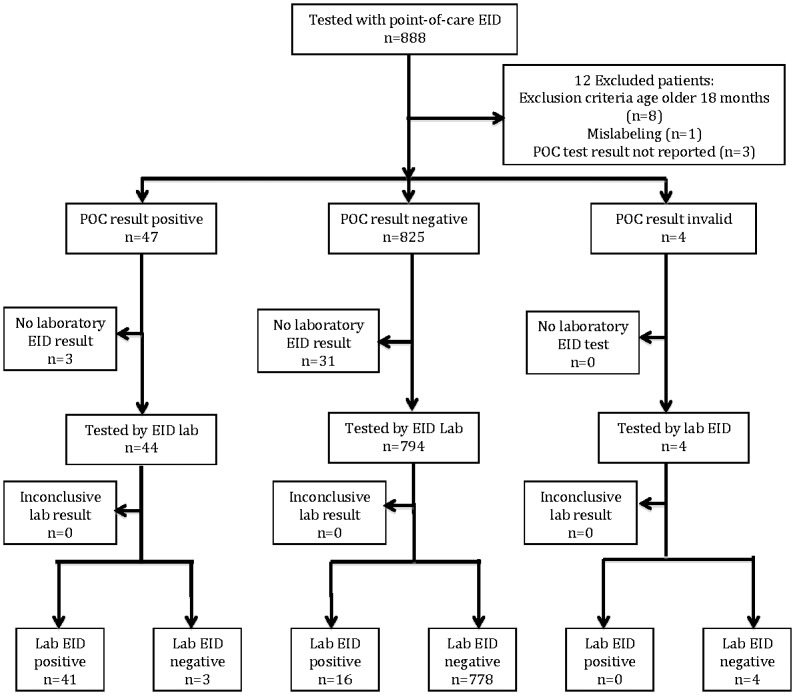
Flow diagram of study participants.

Between May 2013 and March 2014, a total of 888 infants were eligible for the study, with 879 enrolled in the study at the three study site health clinics: 1° de Junho (n = 250), 1° de Maio (n = 252), and Polana Caniço (n = 377) (Fig 1).

Of those enrolled, 838 infants had a valid POC and SOC test result. The POC EID assay had four invalid results with no control line and three records with missing test result data (not reported by the nurse). Thirty-four infants did not obtain a SOC EID test due to poor quality DBS samples (64.7%; n = 22) or lost specimens (35.3%; n = 12). Over half of enrolled infants were female (53.6%; Table 1).

**Table 1:** Demographic and Clinical Characteristics of Mother—Infant Pair Study Participants.

			n = 879	% Total
**Result Lab SOC**	Negative		788	89.6%
	Positive		57	6.5%
	Rejected sample/New Collection		22	2.5%
	Missing result		12	1.4%
**Result POC**	Negative		825	93.9%
	Positive		47	5.3%
	Invalid		4	0.5%
	Missing result		3	0.3%
**Groups**				
		% HIV+^a^	n = 879	% Total
**Health clinics**	CS 1 DE JUNHO	4.4%	250	28.4%
	CS 1 DE MAIO	4.4%	252	28.7%
	CS POLANA CANICO	9.3%	377	42.9%
**Age (in months)**	1–2 months	2.6%	540	61.6%
	2–3 months	9.1%	143	16.3%
	3–6 months	10.1%	129	14.7%
	6–9 months	13.2%	38	4.3%
	> 9 months	44.4%	27	3.1%
**Sex**	Female	7.0%	471	53.6%
	Male	5.9%	408	46.4%
**Mother's prophylaxis**	None	28.6%	14	1.6%
	Option A^b^	16.2%	138	15.7%
	Option B+^c^ (ART)	5.2%	610	69.4%
	Missing Data	5.1%	117	13.3%
**Infant's prophylaxis**	None	46.2%	13	1.5%
	NVP^d^	6.1%	674	76.7%
	NVP + AZT	50.0%	2	0.2%
	AZT^e^	5.3%	19	2.2%
	Missing Data	4.7%	171	19.5%
**Infant's breastfeeding**	No	9.1%	99	11.3%
	Yes	7.1%	548	62.3%
	Missing Data	3.9%	232	26.4%

^a^: HIV status based on the SOC test result.

^b^: WHO recommended set which includes: Antepartum-AZT starting as early as 14 weeks gestation, Intrapartum-at onset of labour, sdNVP and first dose of AZT/3TC, Postpartum- daily AZT/3TC through 7 days postpartum

^c^: Option that include ART as treatment regimen

^d^: Single dose of Nevirapine

^e^: Zidovudine

There was no significant difference in the prevalence of HIV-infection between female and male children (p = 0.488). The majority of infants were between the ages of 1–3 months (77.7%) with a range of 1–18 months. The median age at testing was 42 days (interquartile range 32–70 days), while HIV-negative infants were younger than HIV-infected infants (median age 1.4 vs. 3 months, p<0.001). The proportion of infants who tested positive for HIV with the laboratory-based EID test was 6.7% (n = 57) and this increased with age at testing: 2.7% (95% CI: 1.3–4.1%, n = 14), 9.6% (95% CI: 4.6–14.6%, n = 13), 10.7% (95% CI: 5.1–16.2%, n = 13), 14.3% (95% CI: 2.1–26.5%, n = 5) and 46.2% (95% CI: 25.6–66.7%, n = 12) in the 1–2 months, 2–3 months, 3–6 months, 6–9 months and >9 months age groups, respectively (p<0.001).

Most mothers of the enrolled infants were on triple drug ARV regimens (Option B+) for prevention of mother to child transmission of HIV (PMTCT) (69.4%; n = 610). Only 1.6% of mothers took no prophylaxis (n = 14). PMTCT prophylaxis status was not recorded for 117 mothers (13.3%).

### Performance of POC early infant diagnosis

Of the 838 paired samples tested on both POC and laboratory platforms, a total of 819 concordant samples were identified with 778 negative and 41 positive results on both assays. There were 19 (2.6%) discordant results: three negative and 16 positive by conventional testing. The sensitivity and specificity of the POC EID assay were 71.9% (95% CI: 58.5–83.0%) and 99.6% (95% CI: 98.9–99.9%), respectively (Table 2).

**Table 2:** Performance of the Lynx POC EID p24 Ag test by health care facility and operator.

Health Facility	Operator	Sensitivity	Specificity	Positive Pred. Value	Negative Pred. Value
#1	A	50,0% (3/6)	100,% (130/130)	100,% (3/3)	97,7% (130/133)
	B	0% (0/5)	100% (94/94)	0,0% (0/0)	94,9% (94/99)
	A+B	27.3% (3/11)	100% (224/224)	100% (3/3)	96.5% (223/231)
#2	C	100% (5/5)	99% (114/115)	83,3% (5/6)	100% (114/114)
	D	80,0% (4/5)	100% (105/105)	100% (4/4)	99,0% (105/106)
	E	100%(1/1)	100% (9/9)	100% (1/1)	100%(9/9)
	C+D+E	90.9% (10/11)	99.6% (228/229)	90,9% (10/11)	99.6% (228/229)
#3	F	83,3% (25/30)	99,3% (288/290)	92,6% (25/27)	98,2% (288/293)
	G	60,0% (3/5)	100% (39/39)	100% (3/3)	95,1% (39/41)
	F+G	80.0% (28/35)	99.4% (327/329)	93.3% (28/30)	97.9% (327/334)
TOTAL		71,9%(41/57)	99,6%(779/782)	93,2%(41/44	97,9%(778/794)

The predictive value of a positive test was 93.2% (95% CI: 81.3–98.6%) and the predictive value of a negative test was 97.9% (95% CI: 96.8–98.8%).

The positivity rate differed significantly between the POC and SOC platforms, as shown by McNemar’s test (p = 0.0059). In addition, the conditional probability of both tests producing the same results for positive and negative samples was 81.2% (95% CI: 72.9–89.5%) and 98.8% (95% CI: 98.3–99.3%), respectively.

The sensitivity of the POC EID test varied by operator. The group of operators at Facility #1 had a sensitivity of 27.3%, while the group of operators at Facility #2 had a sensitivity of 90.9% (Table 2). The Pearson’s chi-square test (14.303; p = 0.001) indicated a strong effect on the POC EID p24 Ag test results when operators are grouped by health facility. Two nurses contributed to half (8) of the 16 false negative results. More than half (4) of the operators had a sensitivity greater than or equal to 80.0%, but two operators had a sensitivity of 50% or less. There was no statistically significant association between true positive and false negative p24 assay results and infant age, mother or infant prophylaxis.

### Patient yield grid

**Fig 2:**
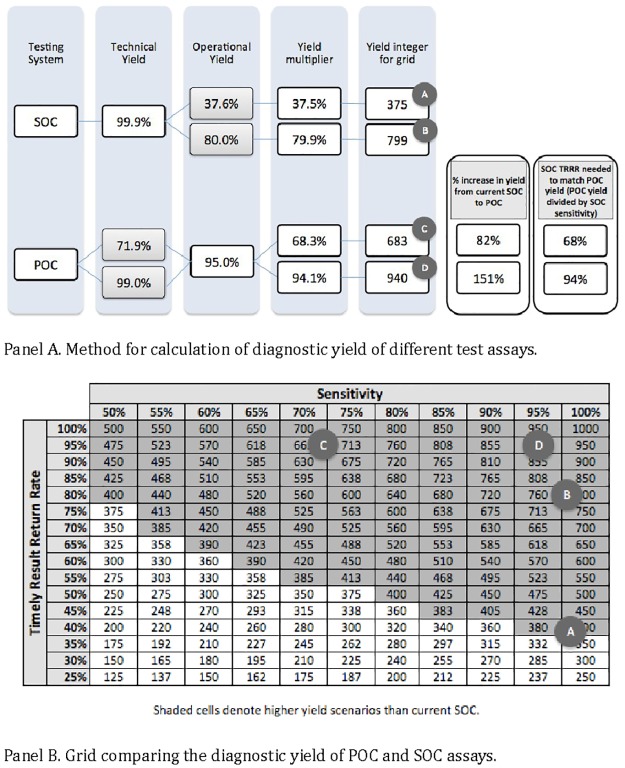
Comparing Technologies for Potential Patient Identification.

We next developed a theoretical algorithm and grid to compare patient yield between diagnostic assays, and inputted data from the POC EID test and the SOC EID testing program, as well as alternative scenarios for comparison (Fig 2).

The standardized algorithm was broadly developed for consideration and use beyond technical performance and test results return. Within our study, we used the sensitivity of each assay as the technical yield multiplied by the test result return rate as the operational yield to generate the yield multiplier. Considering a population of 1,000 HIV-positive infants, the yield multiplier was used to determine the yield calculation or yield integer. The grid maps the intersection of sensitivity and timely result return rates. The potential yield from different technologies can be compared visually by marking their relative position in the grid, such as the scenarios of current SOC EID (circle A), an improved SOC test results return rate system (circle B), the POC EID assay (circle C), and an alternative, better-performing assay (circle D). Shaded cells denote higher yield scenarios than current SOC.

We used a proposed estimate of 95% for timely results observed in POC environments and national EID program data indicating 37.6% of results returned within 28 days for SOC, as recommended by WHO. Circle A identifies the SOC scenario notably characterized by high technological sensitivity but low test return rates, yielding timely results to only 375 of 1,000 theoretically tested infants known to be HIV-positive. At any point within the shaded area, an alternative assay would theoretically have higher yields for identification of infected infants compared to the standard of care. Circle C identifies the POC scenario from this study, with suboptimal sensitivity but higher return rates, yielding timely results to 683 of 1,000 theoretical patients. Our results suggest that the use of the POC EID test could link up to 81% more infected infants to timely care in Mozambique, compared to SOC. Timely test result return rates for current SOC would have to improve to 68%—nearly double the current rate—before patient identification yields would surpass those of this less sensitive POC test. Circle B represents this improved SOC system due to a higher test result return rate (yield of 799 of 1,000 theoretical patients), while Circle D represents an alternative POC assay, with higher sensitivity (yield of 940 of 1,000 theoretical patients).

## Discussion

Access to EID and timely EID results are critical barriers limiting access to ART in HIV-infected infants in low resource settings [1]. Presently, most health systems rely solely on centralized laboratory-based EID that may limit test access in rural communities and have not identified HIV-infected children in numbers required to attain national and global ART targets [12]. Centralized testing for EID in resource-limited settings is also often associated with delays of weeks or months before results are available and a substantial proportion of children are lost to follow up before ART initiation [1,13].

Novel POC technologies have the potential to improve access to diagnosis and linkage to care by providing testing close to patients and reducing results delivery time [13]. Recent studies with nucleic acid testing (NAT) POC technologies for EID have demonstrated high sensitivity and specificity [7,14,15]. However, the cost-effectiveness of implementing NAT POC assays in thousands of EID testing facilities in low-resource countries is unclear, especially when most of these are likely to be low-volume testing sites. For example, in Mozambique, where currently EID is centrally performed in five laboratories, there are potentially over a thousand primary health care facilities that could benefit from POC testing, with more than 90% of these collecting less than 10 EID specimens per week. In this context, low-cost technologies based on immunological principles, such as the one we evaluated here for detecting the HIV Gag p24 antigen, may play a role in efficiently decentralizing EID.

Our results demonstrated that the POC p24 test conducted on 4 week old to 9 months HIV exposed infants had lower sensitivity than the gold-standard laboratory-based NAT assay. This may be due to: i) the p24 antigen being less available for direct detection after anti-HIV antibodies appear in the blood [9]; ii) low levels of p24 antigenemia in a proportion of infected children [9]; iii) the lower efficiency of signal amplification in immunoassays when compared to NAT assays. Furthermore, the sensitivity of the p24 assay varied by health facility and operator, suggesting that, in the current format, the test performance is significantly impacted by human error. The LYNX p24 antigen test comprises several manual steps, with strict time intervals between steps. Moreover, the interpretation of the results is visual and may be challenging if result lines have low density. Nevertheless, four out of the seven operators in the study achieved assay sensitivities above 80%, suggesting that training and adherence to procedures may positively influence test performance. Counter-intuitively, POC tests designed for peripheral health facilities may have to incorporate higher levels of technological sophistication to reduce the number of manual steps.

In most of sub-Saharan Africa, ART in pregnant women is increasingly implemented to prevent the transmission of HIV to newborns. Where successful, this is reducing substantially the number of children born with HIV [1]. In the three health facilities of this study, during a period of 11 months in a city with HIV prevalence, we identified 57 HIV-infected children. This relatively low number of positive subjects affected the confidence interval of the estimated POC assay sensitivity. This issue must be taken into consideration when planning the evaluation of new EID technologies that are in the pipeline.

Evaluations of diagnostic assays and decisions regarding their utility have traditionally focused on analytical performance, most commonly sensitivity, specificity and predictive values, as well as test operational features [16]. However, access to test results is often not considered when comparing technologies or planning their deployment in health systems. This is especially relevant for laboratory networks in resource-limited settings where certain tests are conducted remotely in centralized laboratories and are inefficient in timely test result delivery to patients [1,13]. We used a simple model based on the performance of the POC and laboratory-based technologies in our study to compare the effective availability of test results for HIV-infected children between a new POC assay and the gold standard laboratory-based test. Our results demonstrate that, compared with remote laboratory testing with slow results return and rates of loss-to-follow-up, on-site testing returned a greater proportion of results to mother-infant pairs. This helped offset the suboptimal sensitivity of the POC assay, resulting in an overall greater public health impact, with more HIV-positive children identified and potentially linked to timely care [12]. Although these theoretical assumptions may benefit from further research and ethical discussions, we envisage that POC EID assays that are easy to operate and feasible for remote locations, but which perform below classically accepted thresholds may play an important role in increasing access to timely diagnosis and life saving ART in resource-limited settings.

## Supporting Information

S1 Dataset Clinical study database. (XLS)
